# The *Drosophila* nucleoporin ELYS is required for parental chromosome arrangement at fertilization

**DOI:** 10.1093/g3journal/jkaf104

**Published:** 2025-05-13

**Authors:** Kazuyuki Hirai, Hiroki Sakamoto, Yoko Keira, Mika Ozaki, Kanta Yamazoe, Hiroyuki O Ishikawa, Yoshihiro H Inoue, Kyoichi Sawamura

**Affiliations:** Department of Biology, Kyorin University School of Medicine, Mitaka, Tokyo 181-8611, Japan; Graduate School of Life and Environmental Sciences, University of Tsukuba, Tsukuba, Ibaraki 305-8572, Japan; Graduate School of Science, Chiba University, Chiba, Chiba 263-8522, Japan; Biomedical Research Center, Kyoto Institute of Technology, Kyoto, Kyoto 606-8585, Japan; Biomedical Research Center, Kyoto Institute of Technology, Kyoto, Kyoto 606-8585, Japan; Graduate School of Science, Chiba University, Chiba, Chiba 263-8522, Japan; Biomedical Research Center, Kyoto Institute of Technology, Kyoto, Kyoto 606-8585, Japan; Faculty of Life and Environmental Sciences, University of Tsukuba, Tsukuba, Ibaraki 305-8572, Japan

**Keywords:** centrosomes, early embryos, maternal effects, spindle bipolarity, pronuclear apposition, the first mitosis, tripolar spindles, zygotes

## Abstract

One key aspect of fertilization is the unification of the maternal and paternal genomes driven by the first mitotic spindle. However, little is known about the mechanisms that underlie the formation of a bipolar spindle that interacts with the two discrete chromosome sets in juxtaposition. We here show that, in *Drosophila*, the maternally provided ELYS—an evolutionarily conserved subunit of the nuclear pore complex—localizes to female and male pronuclei and then redistributes to the interior of the spindle and the resulting zygotic nuclei. Both *Elys* loss-of-function mutations and ELYS overexpression in the female germline were associated with maternal-effect lethality. Our cytological studies of fertilized eggs revealed that ELYS is primarily involved in the apposition of female and male pronuclei, potentially impacting the parental genome configuration of the first mitotic spindle. We propose that pronuclear apposition is essential for centrosome localization at the emergent pronuclear junction to promote bipolar spindle formation for the first mitosis. In addition, we discuss the possible involvement of ELYS in interspecific hybrid incompatibility.

## Introduction

In the newly fertilized egg, the maternal and paternal genomes are confined in their respective pronuclei, which individually enter the first cell cycle. In many animal species, the parental chromosomes align separately at the equator of the first mitotic spindle and zygotic nuclei are created during late mitosis, with both parental sets of separated chromatids being packaged for the first time within a common nuclear membrane (Henking 1892; Huettner 1924; Kawamura 1978; Sato and Tanaka-Sato 2002; Tram *et al.* 2006; Lindeman and Pelegri 2012; Reichmann *et al.* 2018; Xu *et al.* 2019; Rahman *et al.* 2020; Schneider *et al.* 2021). In recent years, the importance of the first mitotic division has been appreciated for fertilization success in humans (McCoy *et al.* 2015; Currie *et al.* 2022; Ono *et al.* 2024; Porokh *et al.* 2024). However, the fundamental nuclear and cytoplasmic mechanisms underlying the arrangement of parental sets of chromosomes for the first mitosis remain largely unknown.

In *Drosophila*, the female and male pronuclei are initially positioned nearer the egg cortex and at the center of the egg, respectively. The centrosomes accompanying the male pronucleus form the sperm aster, and, guided by the microtubules, the female pronucleus migrates towards the male pronucleus, giving rise to pronuclear apposition. Importantly, a microtubule-based bipolar mitotic spindle forms that interacts with the two pronuclei in juxtaposition (reviewed in Foe *et al.* 1993; Liu *et al.* 1997; Loppin *et al.* 2015; Blake-Hedges and Megraw 2019). We have recently shown that the ELYS protein plays a crucial role during the separation of the neighboring maternal and paternal genomes at fertilization. Females mutant for the *Elys* gene produce embryos that arrest development during metaphase of the first mitosis, where a faulty mitotic spindle forms around the parental chromosomes that are abnormally coalesced into a single mass (Hirai *et al.* 2018). ELYS is one of the subunits of the multiprotein assembly, nuclear pore complex (NPC), embedded in the nuclear envelope of eukaryotic cells, mediating essential cellular functions such as selective transport of molecules between the nucleoplasm and cytoplasm and nuclear and cytoskeletal organization (Buchwalter *et al.* 2019; Guglielmi *et al.* 2020; Shevelyov 2020; Kutay *et al.* 2021; Makarov *et al.* 2021; Raices and D'Angelo 2022). However, at least in *Drosophila*, *Elys* loss-of-function alleles allow the mutant individuals to survive with no discernible defects but cause abnormal embryonic progeny. Thus, ELYS is essential for the earliest developmental process that is exclusively executed by maternal gene products deposited in the oocyte (Vastenhouw *et al.* 2019; Thomalla and Wolfner 2025), although the protein appears to have important functions during various developmental stages (Kuhn *et al.* 2019; Mehta *et al.* 2020).

In the present study, we confirmed the functional role of ELYS at fertilization by using inducible transgenic rescue constructs in *Elys* null mutant females. We also report the effect of ELYS overexpression in the germline of wild-type females, resulting in the formation of a tripolar, but not bipolar, spindle that separately surrounds sets of parental chromosomes. Our cytological analyses revealed that the primary defect associated with both loss- and gain-of-function of ELYS is in pronuclear apposition in preparation for the first mitosis. The present study provides new insights into the functional role for a nucleoporin in pronuclear coordination essential to create a zygote.

From an evolutionary point of view, we have also examined the effect of ectopic expression of ELYS from *Drosophila simulans*, a closely related species of *D*. *melanogaster*, and discuss the results with respect to previous studies suggesting the possible involvement of NPC components in hybrid incompatibility (Presgraves *et al.* 2003; Tang and Presgraves 2009; Sawamura *et al.* 2010; Hirai *et al.* 2018).

## Materials and methods

### Fly strains

The wild-type strains used in the present study were *D*. *melanogaster* Oregon-R and *D*. *simulans* C167.4, showing high egg hatchability at 89.5 and 86.0%, respectively (*n* = 200). The details of the *D*. *melanogaster Elys*^2^ and *Elys*^5^ alleles have been described previously (Hirai *et al.* 2018). The alleles are functionally null (thus designated as *Elys*^−^ hereafter) and kept as *y*^2^  *cho*^2^  *v*^1^  *Elys*^–^/*FM7c* strains. The other fly strains used are described below. To avoid the interference of endosymbiotic bacteria (presumably *Wolbachia*) during the phenotypic analysis of embryos, we eliminated them from the stocks by growing flies with medium containing 0.03% tetracycline for one generation (Lin and Wolfner 1991). Flies were reared at 25°C. All strains used in this study are listed in Reagent Table. We used FlyBase to find information on stocks, phenotypes, genes, and proteins (Öztürk-Çolak *et al.* 2024).

### Establishment of *Elys* transgenes

For PCR, we used PrimeSTAR Max DNA Polymerase or Tks Gflex DNA Polymerase (Takara Bio). To create *UASp*-*Elys*^mel^, full-length *Elys* of *D*. *melanogaster* (*Elys*^mel^; 6,336 bp) was PCR-amplified from cDNA clone LD14710 (Rubin *et al.* 2000) using primers 5′-ATC AGA TCC GCG GCC GCA TGG AGT GGC ACG-3′ and 5′-GTG GCC TAT GCG GCC GCC TAA TGC TCC GAC-3′, and the resulting fragment was cloned into *Not*I-cut pUASPattB using the Seamless Ligation Cloning Extract (SLiCE) method (Motohashi 2015). To create *UASp*-*Elys*^mel^-*mCherry*, *Elys*^mel^ excluding the stop codon was PCR-amplified from *UASp*-*Elys*^mel^ using primers 5′-ATC AGA TCC GCG GCC GCA TGG AGT GGC ACG AAG TG-3′ and 5′-GCC GGA TCC ACC GCT ATG CTC CGA CTT GGA GGT-3′. A fragment including mCherry with an *N*-terminal flexible 4 × GlyGlySer linker was PCR-amplified from *UAS*-*fj*-*mCherry* (H.O.I., unpublished) using primers 5′-AGC GGT GGA TCC GGC GGT-3′ and 5′-GTG GCC TAT GCG GCC GCT TAC TTG TAC AGC TCG TC-3′, and then the two PCR fragments were cloned into *Not*I-cut pUASPattB using the SLiCE method.

To create *UASp*-*Elys*^sim^, full-length *Elys* of *D*. *simulans* (*Elys*^sim^; 6,159 bp) was cloned from a C167.4 strain cDNA pool. Total RNA was extracted from the adult flies using ISOSPIN Cell & Tissue RNA (NIPPON GENE), and cDNA was synthesized from the total RNA using Super Script IV VILO Master Mix (Thermo Fisher Scientific). Two segments of *Elys*^sim^ were PCR-amplified from the cDNA pool using primers pairs (5′-ATC AGA TCC GCG GCC GCA TGG AGT GGC ACG-3′ and 5′-CAT CTT TGA AGG GGA ATC GCA TTG TGT G-3′) and (5′-TCC CCT TCA AAG ATG ACC CCC GTT TC-3′ and 5′-GTG GCC TAT GCG GCC GCC TAA TGC TCC GAC-3′), and the two resulting fragments were cloned into *Not*I-cut pUASPattB using NEBuilder HiFi DNA Assembly (New England BioLabs). To create *UASp*-*Elys*^sim^-*mCherry*, *Elys*^sim^ excluding the stop codon was PCR-amplified from *UASp*-*Elys*^sim^ using primers 5′-ATC AGA TCC GCG GCC GCA TGG AGT GGC ACG AAG TG-3′ and 5′-GCC GGA TCC ACC GCT ATG CTC CGA CTT GGA GGT-3′. The PCR fragments of *Elys*^sim^ and mCherry with a linker used for the *UASp*-*Elys*^mel^-*mCherry* cloning were cloned into *Not*I-cut pUASPattB using NEBuilder HiFi DNA Assembly. The cloned *Elys*^mel^ and *Elys*^sim^ were re-sequenced to confirm the absence of unwanted mutations. We used both tagged and nontagged transgenes in the present analyses, and there were no essential differences between them (see text).

The constructs were each injected into embryos from *D*. *melanogaster* strain *y*^1^  w* *P*{*nanos*-*phiC31*\*int*.*NLS*}X; *P*{*Cary*}*Msp300*^attP40^ to allow for phiC31-targeted, site-specific recombination into the attP landing site (cytological position 25C6 on chromosome *2*L) (Bischof *et al.* 2007). Transgenic offspring were screened for w^+^ eye color. The original transgenes were in the genetic background of *Elys*^+^, but later the genetic background was replaced with the *Elys*^2^ or *Elys*^5^ allele via conventional backcrosses using chromosome *X* and *2* balancers. Transgene expression was driven by the GAL4/UAS system. The *Gal4 s*trains used were w*; *P*{*matα4*-*GAL*-*VP16*}V2H and w*; *P*{*GAL*-*nanos*.*NGT*}40 (Hudson and Cooley 2014). They are hereafter abbreviated as *matα*-*Gal4* and *nos*-*Gal4*, respectively. The strains were crossed with the *Elys* (*Elys*^mel^ or *Elys*^sim^) transgenic strains, and females trans-heterozygous for *Gal4* and *UASp*-*Elys* or *UASp*-*Elys*-*mCherry* were used for the experiments. The genotypes are abbreviated as *matα*-*Gal4* > *Elys*, for example. The maternally expressed *Elys* transgenes were confirmed by quantitative reverse transcription-PCR (qRT-PCR).

### Quantitative reverse transcription-PCR

Virgin females of the target genotypes were collected and allowed to mature for 7 days. Ovaries from five flies with each genotype were collected and stored in TRIzol Reagent (Invitrogen). Total RNA was extracted from ovaries using the reagent, and cDNA was synthesized from the total RNA using the PrimeScript II first strand cDNA Synthesis kit (Takara Bio) with an oligo dT primer. qRT-PCR was performed using the FastStart Essential DNA Green Master (Roche) and a Light Cycler Nano Instrument (Roche). Amplification curves were generated for the following reaction: initial denaturation step at 95°C for 5 min, followed by 45 cycles at 95°C for 5 s, 60°C for 5 s, and 72°C for 8 s. To generate melting curves, the fluorescence signals were recorded continuously from 60°C to 95°C as the temperature was increased at a rate of 0.1°C/sec. The *Elys* mRNA was quantified (Supplementary Figs. 1 and 2; see the legends for the primers). Each sample was PCR-amplified in duplicate, and the results from those replicates were averaged. For quantification, the ΔΔCt method was used to determine the differences in the expression of target genes relative to the expression of the reference gene *Rp49*.

### Egg-laying and egg hatchability assay

Well-fed virgin females of various genotypes were mated with males of the wild-type strain of the same species and allowed to lay eggs on fly medium on which yeast was seeded. Eggs were collected within 6 h after deposition and aligned on the 1.5% agar plate; after ∼24 h both hatched and nonhatched eggs were counted.

### Embryo collection, immunostaining, and imaging

To examine embryonic phenotypes, laid embryos were collected at 20-min intervals and fixed and stained with below-mentioned antibodies as described previously (Hirai *et al.* 2023). Antibodies were raised in rabbits against a synthetic peptide corresponding to the most C-terminal 14 amino-acid residues (ELRPRLRRTSKSEH) of ELYS from both *D*. *melanogaster* and *D*. *simulans*, and the antibodies were affinity purified against the immunogenic peptide (Eurofin Genomics). Other primary antibodies used were rat monoclonal anti-Tubulin (YL1/2, 1:300; Abcam), rabbit anti-ELYS (1:300; this study), rabbit anti-DsRed (1:300; Clontech), rabbit anti-Centrosomin (1:3,000; Lucas and Raff 2007), and guinea pig anti-Asterless (1:3,000; Roque *et al.* 2012). All secondary antibodies were diluted 1:800 [Alexa Fluor 488–conjugated goat antirat IgG (Thermo Fisher Scientific), Cy3-conjugated AffiniPure goat antirabbit IgG (Jackson ImmunoResearch) and Alexa Fluor 647-conjugated goat antiguinea pig IgG (Thermo Fisher Scientific)]. Anti-DsRed was used to detect mCherry, as mCherry fluorescence was not preserved in fixed embryos. DNA was stained with DAPI. Samples were mounted in Fluoro-KEEPER antifade reagent (Nacalai Tesque) and observed on a FLUOVIEW FV1000 fluorescence microscope with a 60×/1.30 Sil UPlanSApo objective (Olympus). Images were acquired as *z*-series at 0.5-μm intervals with the laser power and gain settings determined for each embryo and then processed as maximum-intensity projections using ImageJ (NIH) and Photoshop (Adobe).

### Data visualization and statistical tests

All data graphs were generated in Prism 7 (GraphPad Software). Statistical tests were conducted by using R4.1.2 (R-Project).

## Results

### Subcellular localization of the nucleoporin ELYS in early stage embryos

**Fig. 1.**
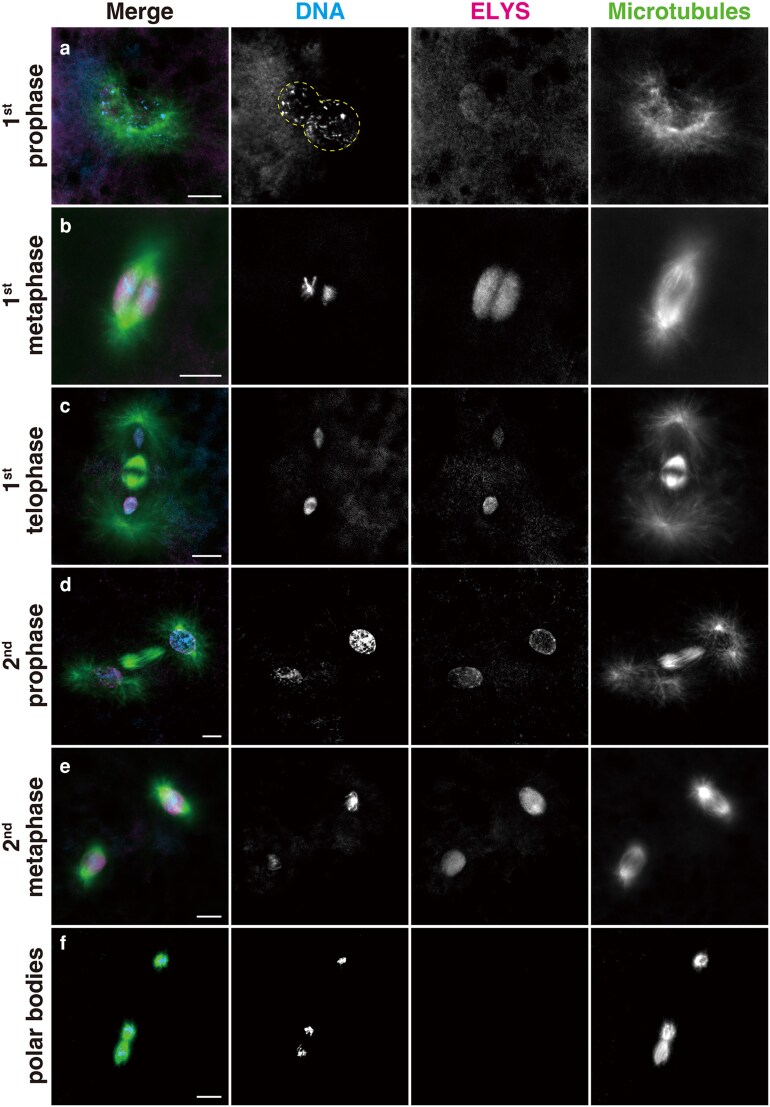
Localization of ELYS during the first and second mitotic divisions in *Drosophila melanogaster*. Embryos were fixed and stained with the DNA dye DAPI and antibodies specific for ELYS and α-tubulin (for microtubules). The superimposed staining of ELYS and microtubules appears white. a) First prophase: The female and male pronuclei are arranged and contact with each other. The edge of the pronuclei is outlined with dashed lines for clarity. ELYS labeling is faint in the nucleoplasm. b) First metaphase: The spindle consists of two halves of microtubule arrays, each encompassing a parental set of replicated chromosomes. ELYS is broadly distributed between the poles of each microtubule unit. c) First telophase: ELYS relocalizes to the daughter nuclei containing both parental sets of separated chromatids. d) Second prophase: ELYS spreads across the pronuclei. e) Second metaphase: ELYS is present broadly within the spindle region between the poles. f) ELYS is not detectable around the pole bodies. In panels (c) and (d), one of the nuclei appears brighter than the other because of the difference in depth resulting from the tilted axis of the cleavage division. Scale bars, 10 μm.

The extremely rapid *Drosophila* fertilization process culminates in the production of two zygotic nuclei during telophase of the first mitosis within 15 min after egg deposition (Foe *et al.* 1993). During early stages of the first mitosis, the two groups of replicated parental chromosomes remain separated but still close to each other (Fig. 1, a and b). However, in embryos produced by *Elys* loss-of-function (*Elys*^–^) mutant females, the two sets of chromosomes are abnormally fused in a mass and development arrests in a metaphase-like state of the first mitosis (Hirai *et al.* 2018). To understand the basis for this abnormal phenotype, we first determined the normal localization of ELYS in embryos of the wild-type strains using polyclonal antibodies recognizing ELYS. In agreement with the role of the maternally derived ELYS (Hirai *et al.* 2018), ELYS labeling became intense after meiotic completion at all four meiotic products but was undetectable around the highly condensed sperm nucleus (Supplementary Fig. 3a), i.e. before its conversion to the male pronucleus. During pronuclear migration, ELYS appeared to surround the region of both female and male pronuclei (Supplementary Fig. 3b), with lesser nucleoplasmic localization. Thereafter, during prophase of the first mitosis, ELYS showed faint but consistent localization in the interior of both pronuclei in juxtaposition (Fig. 1a).

Along the first mitotic spindle, parental chromosomes stay as separate maternal and paternal genomes in discrete regions of the metaphase plate. ELYS showed characteristic bi-lobed distribution, each surrounding a set of parental chromosomes, in the region between the opposite poles (Fig. 1b). The spindle areas labeled with ELYS probably corresponded to the region delimited by the spindle envelope of the semi-open mitosis (Stafstrom and Staehelin 1984; Callaini and Riparbelli 1996; Schweizer *et al.* 2015). ELYS then redistributed to the daughter nuclei containing both sets of parental chromatids (Fig. 1, c and d). During the subsequent mitotic divisions, ELYS was widespread in the metaphase spindle surrounding the diploid sets of chromosomes (Fig. 1e). ELYS was not detected in polar bodies lying near the cortex of the egg (Fig. 1f).

### ELYS impacts the parental genome configuration of the first mitotic spindle

*Elys* ^–^ induces fully penetrant maternal-effect lethality of the embryonic progeny that is not rescuable by fertilization with *Elys*^+^ sperm (Hirai *et al.* 2018). We performed rescue experiments by expressing a transgene encoding *Elys*^+^ or *Elys*  ^+^ -*mCherry* (the transgenic *Elys*^+^ is designated as *Elys*^mel^ hereafter) in the germline of *Elys*^–^ mutant mothers under GAL4/UAS control using two different germline-specific promoters (Peel *et al.* 2007; Gilliland *et al.* 2024; Haseeb *et al.* 2024). The maternal-effect embryonic lethality caused by *Elys*^–^ was partially rescued by *Elys*^mel^ expression by using the *nos*-*Gal4* or *matα*-*Gal4* driver, with the latter more effective than the former (Table 1; Supplementary Table 1 and Fig. 1a).

**Table 1.** Development of embryos produced by *Elys*^−^ females expressing *Elys*^mel^ transgenes.

Maternal genotype		Hatchability (%)
*Elys* locus	Transgene	
*Elys* ^+^	—	89.5
*Elys* ^–^	—	0
*Elys* ^–^	*nos*-*Gal4* > *Elys*^mel^	11.5
*Elys* ^–^	*nos*-*Gal4* > *Elys*^mel^-*mCherry*	7.0
*Elys* ^–^	*matα*-*Gal4* > *Elys*^mel^	33.5
*Elys* ^–^	*matα*-*Gal4* > *Elys*^mel^-*mCherry*	40.5

Females of the indicated genotypes were crossed with wild-type males, and egg hatchability was examined (*n* = 200). Exogenous *Elys* expression was driven by female germline GAL4 (*nos*-*Gal4* or *matα*-*Gal4*). *Elys*^–^: the functionally null *Elys*^5^ allele. The driving effect is significantly larger in *matα-Gal4* than in *nos-Gal4*: *Elys*^5^; *nos-Gal4* > *Elys*^mel^  *vs*. *Elys*^5^; *matα-Gal4* > *Elys*^mel^ (*χ*^2^ = 26.509, d.f. = 1, *P* = 2.623E-7), *Elys*^5^; *nos-Gal4* > *Elys*^mel^-*mCherry vs*. *Elys*^5^; *matα-Gal4* > *Elys*^mel^-*mCherry* (*χ*^2^ = 60.135, d.f. = 1, *P* = 8.859E-15).

**Fig. 2.**
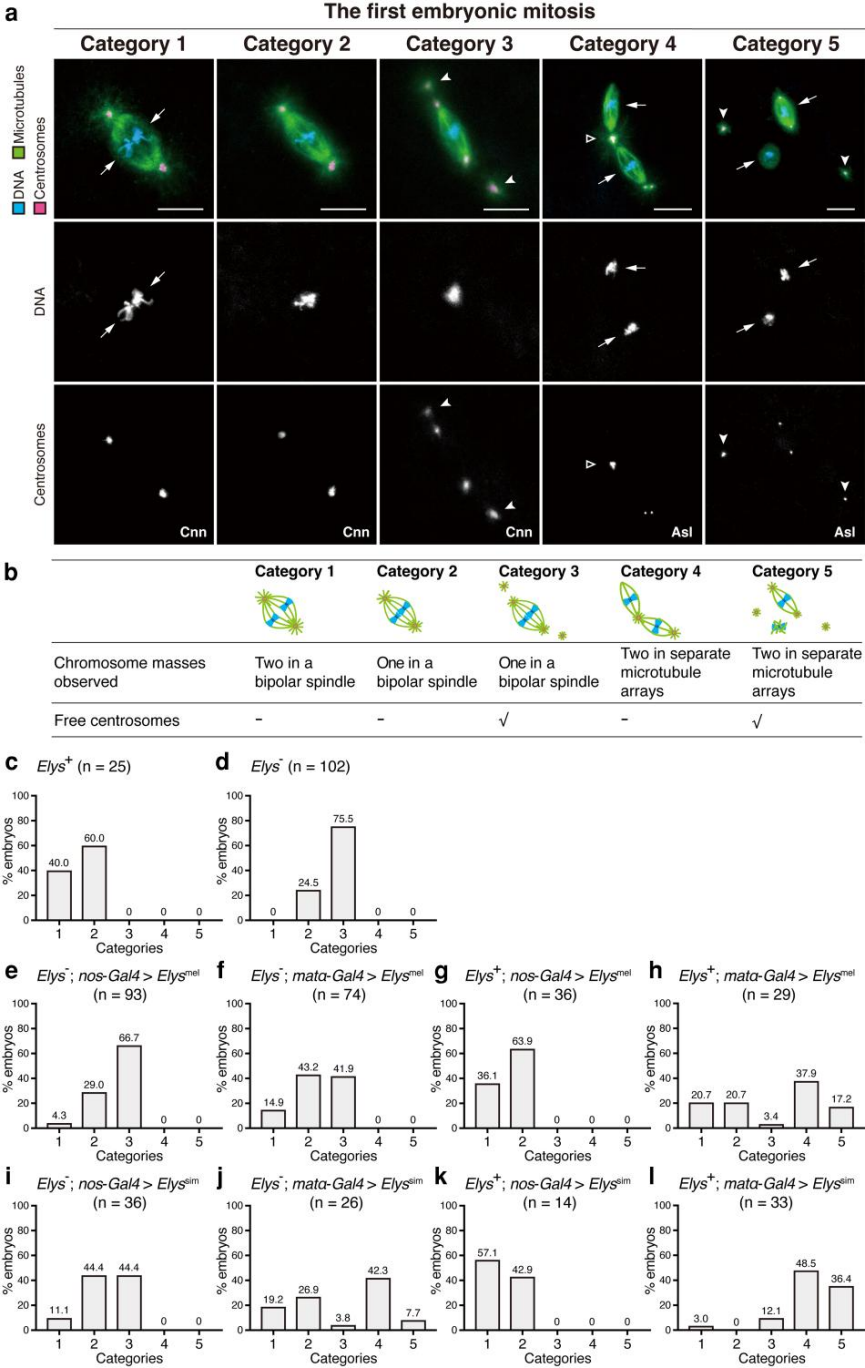
Abnormal configurations of the first mitotic spindle induced by inadequate or excessive ELYS. Embryos were fixed and stained with the DNA dye DAPI and antibodies against α-tubulin for microtubules and either Centrosomin (Cnn) or Asterless (Asl) for centrosomes. a and b) Mitotic figures during metaphase of the first embryonic mitosis were assigned to five categories. Category 1: A bipolar spindle is organized around two discrete sets of parental chromosomes in juxtaposition (arrows). The centrosomal aster is situated at each spindle pole. Category 2: A bipolar spindle forms around one mass of parental chromosomes. The visual appearance of parental chromosomes can reflect either the angle from which two separate sets of parental chromosomes are viewed as one mass or abnormal fusion of both chromosome sets into a mass; the two states are not distinguishable from each other. Category 3: It is similar to category 2, except the presence of centrosomes that are individualized and detached from the spindle poles (free centrosomes; arrowheads). Categories 4 and 5: Two microtubule arrays spindles—each encompassing a parental set of chromosomes—are either linked at the central poles with a shared aster (outlined triangle), resulting in a tripolar spindle, or completely separated. All centrosomes are located at spindle poles in category 4. Category 5 is characterized by the presence of free centrosomes in the cytosol (arrowheads). c–l) The graphs quantify the five mitotic configuration categories in embryos produced by females of the indicated genotypes. c) Wild-type. d) *Elys*^−^. Data are from Hirai *et al.* (2018). e–h) Transgenic expression of the *Drosophila melanogaster* version of ELYS. e) *Elys*^−^; *nos-Gal4*> *Elys*^mel^-*mCherry*. f) *Elys*^−^; *matα-Gal4*> *Elys*^mel^-*mCherry*. g) *Elys*^+^; *nos-Gal4*> *Elys*^mel^-*mCherry*. h) *Elys*^+^; *matα-Gal4*> *Elys*^mel^ and *Elys*^+^; *matα-Gal4*> *Elys*^mel^-*mCherry*. i–l) Transgenic expression of the *Drosophila simulans* version of ELYS in *D*. *melanogaster*. i) *Elys*^−^; *nos-Gal4*> *Elys*^sim^-*mCherry*. j) *Elys*^−^; *matα-Gal4*> *Elys*^sim^-*mCherry*. k) *Elys*^+^; *nos-Gal4*> *Elys*^sim^-*mCherry*. l) *Elys*^+^; *matα-Gal4*> *Elys*^sim^-*mCherry*.

We then cytologically assessed spindle phenotypes associated with *Elys*^-^. In our analysis of the first mitotic spindle, 40% of the embryos produced by wild-type females showed two discrete masses of chromosomes in a bipolar spindle (category 1; Fig. 2, a–c) as expected for the normal first mitotic spindle encompassing the two separate parental chromosome groups (Fig. 1b). In the remaining embryos, however, only one mass of mitotic chromosomes was observed in a bipolar first mitotic spindle (category 2, Fig. 2, a–c), probably reflecting the angle from which two adjacent sets of parental chromosomes are viewed. In embryos produced by *Elys*^-^ females, no first mitotic spindle was classified as category 1. Instead, when ELYS is missing, the parental chromosomes were found in a single mass in the first mitotic spindle of the embryo (categories 2 and 3). Besides the spindles showing normal centrosome localization at the spindle poles (category 2), the majority of the mutant embryos showed abnormal centrosome behavior; centrosomes are precociously individualized and detached from the spindle poles into the cytosol (category 3) (Fig. 2, a, b, and d). When expression of ELYS^mel^ was driven in the germline of *Elys*^-^ females by *nos*-*Gal4* (Fig. 2e) or *matα*-*Gal4* (Fig. 2f), there was an increase in the proportions of categories 1 and 2 and a decrease in that of category 3. The rescue of the *Elys*^-^ phenotype with the ELYS^mel^ transgenes, albeit partially, confirms the role of ELYS in parental genome configuration of the first mitotic spindle.

We next overexpressed ELYS using *nos*-*Gal4* > *Elys*^mel^ and *matα*-*Gal4* > *Elys*^mel^ in the germline of wild-type females. The weak *nos*-*Gal4* driver had no discernible effect on egg hatchability (Table 2) and the first mitotic spindle (Fig. 2g). However, overexpression using the *matα*-*Gal4* driver caused obvious reduction in egg hatchability (Table 2). Furthermore, the mitotic phenotype of increased amounts of ELYS was different from that caused by *Elys*^-^. In the embryos, parental sets of chromosomes were not adjacently situated and the two microtubule arrays surrounding them were either tandemly aligned with a shared centrosome at the central poles, showing a tripolar configuration, or spatially separate (categories 4 and 5, Fig. 2, a, b, and h). Categories 4 and 5 were separated by the absence or the presence of free centrosomes in the cytosol. Taken together, these results suggest that ELYS protein levels impact the arrangement of parental sets of chromosomes and organization of the first mitotic spindle in fertilized eggs.

**Table 2.** Overproduction of ELYS is toxic to embryonic development.

Maternal genotype		Hatchability (%)
*Elys* locus	Transgene	
*Elys* ^+^	*Elys* ^mel^	90.0
*Elys* ^+^	*matα*-*Gal4*	94.5
*Elys* ^+^	*nos*-*Gal4* > *Elys*^mel^	89.0
*Elys* ^+^	*nos*-*Gal4* > *Elys*^mel^-*mCherry*	94.0
*Elys* ^+^	*matα*-*Gal4* > *Elys*^mel^	47.5
*Elys* ^+^	*matα*-*Gal4* > *Elys*^mel^-*mCherry*	37.5

Females of the indicated genotypes were crossed with wild-type males, and egg hatchability was examined (*n* = 200). Exogenous *Elys*^mel^ expression was driven by the female germline GAL4, *nos-Gal4* or *matα*-*Gal4*. The driving effect is significantly larger in *matα-Gal4* than in *nos-Gal4*: *Elys*^+^; *nos-Gal4* > *Elys*^mel^  *vs*. *Elys*^+^; *matα-Gal4* > *Elys*^mel^ (*χ*^2^ = 77.575, d.f. = 1, *P* < 2.2E-16), *Elys*^+^; *nos-Gal4* > *Elys*^mel^-*mCherry vs*. *Elys*^+^; *matα-Gal4* > *Elys*^mel^-*mCherry* (*χ*^2^ = 139.26, d.f. = 1, *P* < 2.2E-16).

### ELYS is primarily involved in apposition of the female and male pronuclei prior to the assembly of the first mitotic spindle

**Fig. 3.**
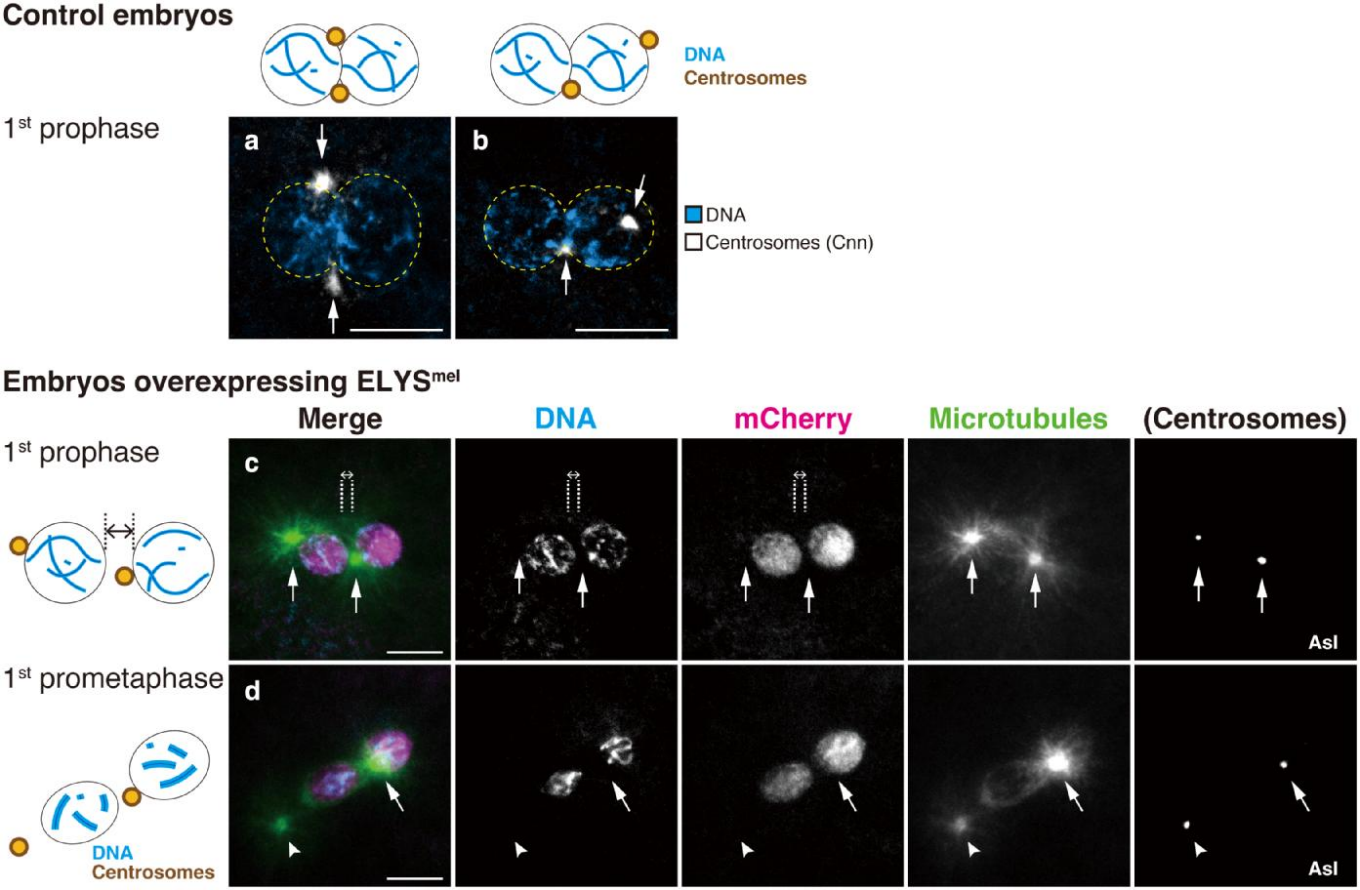
Overexpressing ELYS causes incomplete pronuclear apposition and abnormal centrosome positioning early during formation of the first mitotic spindle. a and b) Control embryos were fixed and stained with the DNA dye DAPI and an antibody specific for a centrosome marker Centrosomin (Cnn). The two-color images on a black background represent normal pronuclear apposition during prophase of the first mitosis. The edge of the adjoining pronuclei is outlined with dashed lines in yellow. a and b) All embryos showed two centrosomes (arrows) attached to the pronuclei (*n* = 51). Both centrosomes reside at the pronuclear junction (a, 41.2%), or one is located at the junction, whereas the other at a different location (b, 58.8%). In (b), one centrosome appears inside the nucleus because of the location on the surface of the spherical structure. c and d) Embryos overexpressing ELYS^mel^-mCherry were fixed and stained with the DNA dye DAPI and antibodies specific for DsRed for ELYS-mCherry (mCherry), α-Tubulin (for microtubules), and a centrosome marker Asterless (Asl; not overlaid in merged images but shown independently). The embryos show a high concentration of ELYS^mel^-mCherry in the pronuclei. c) Two pronuclei are spaced apart. One of the centrosomes (arrows) is in the gap between the pronuclei (parallel dashed lines) and the other at the opposite side of one pronucleus. Note the growing asters from the centrosomes. d) During the first mitotic prometaphase, the two groups of condensed parental chromosomes are separately surrounded by ELYS as well as microtubules. The central centrosome (arrow) generates radial arrays of microtubules that extend to both chromosome groups. The other centrosome (arrowhead) is a short distance away from the opposite side of the left pronucleus. Scale bars, 10 μm. Maternal genotypes: (a and b) the wild-type and *Elys*^+^; *nos*-*Gal4*> *Elys*^mel^-*mCherry* and (c and d) *Elys*^+^; *matα*-*Gal4*> *Elys*^mel^-*mCherry*.

To determine the nature of the aberrant first mitotic spindle caused by ELYS overexpression, we followed the behavior of the pronuclei and associated centrosomes at earlier mitotic stages. Prophase of the first mitosis is marked by the gradual condensation of individual chromosomes from the chromatin mass in the pronuclei (Fig. 1a). In this stage, control embryos produced by females showing normal hatchability of the embryonic progeny (the wild-type and *Elys*^+^; *nos*-*Gal4* > *Elys*^mel^-*mCherry*) invariably showed apposed pronuclei with which two centrosomes were associated (Fig. 3, a and b, *n* = 51). The centrosomes resided at both sides of the pronuclear junction in 41.2% of the embryos (Fig. 3a), whereas, in the remaining embryos, one was located at the junction and the other at a different position on the surface of a pronucleus (Fig. 3b). Embryos produced by females overexpressing ELYS accumulated an abundance of the protein in the interior of the pronuclei (Fig. 3, c and d; *Elys*^+^; *matα*-*Gal4*> *Elys*^mel^-*mCherry*). Pronuclear migration over long distance appeared to have occurred but the two pronuclei lacked direct contact. One of the centrosomes was always located in the space between the slightly separated pronuclei, whereas the other was at a different site of one of the pronuclei (Fig. 3c). In the subsequent prometaphase, microtubules were organized around both pronuclei (Fig. 3d). In the developing spindle, the distal centrosome was sometimes detached in early mitosis (arrowhead in Fig. 3d). The frequencies of incomplete apposition (42.9%; *n* = 7) *vs*. the formation of a tripolar or separated spindle (55.1%; categories 4 and 5 in Fig. 2h; *n* = 29) were almost in agreement. Overall, the results suggest that ELYS protein levels could be related to pronuclear coordination at the spindle and chromosome level.

### Effects of the *Drosophila simulans* version of ELYS in *D*. *melanogaster*

We previously proposed a possible involvement of the nucleoporin ELYS in hybrid incompatibility between *D*. *melanogaster* and its closely related species *D*. *simulans* (Hirai *et al.* 2018). Localization of *D*. *simulans* ELYS (ELYS^sim^) during the first mitosis in *D*. *simulans* embryos (Supplementary Fig. 4) was similar to that of ELYS in *D*. *melanogaster* (Fig. 1). We then performed ectopic expression studies of ELYS^sim^ in *D*. *melanogaster* in the same manner as above mentioned for ELYS^mel^. Expression of ELYS^sim^ via the *nos*-*Gal4* driver partially rescued the maternal-effect embryonic lethality induced by *Elys*^-^ (Supplementary Table 2) and the abnormal spindle phenotype (Fig. 2i), as is the case with ELYS^mel^ (Table 1 and Fig. 2e; Supplementary Table 1). Compared with ELYS^mel^, ELYS^sim^ appeared to be more effective in the rescue experiments. ELYS^sim^ expression via the *matα*-*Gal4* driver in *Elys*^−^ females produced abnormal mitotic spindles classified as categories 4 and 5 (Fig. 2j), which was not the case for ELYS^mel^ (Fig. 2f). Such different phenotypic effect of ELYS^sim^ relative to ELYS^mel^ in *D*. *melanogaster* embryos was also seen in ELYS overexpression studies in *Elys*^+^ females (Fig. 2h for ELYS^mel^  *vs*. Fig. 2l for ELYS^sim^). It is however important to note that the comparative analysis between ELYS^mel^ and ELYS^sim^ solely depends on the hypothesis that ELYS^mel^ and ELYS^sim^ proteins are present at comparable levels in embryos derived from females of otherwise identical genotypes (cf. Supplementary Fig. 2 for mRNA measurements).

## Discussion

### Developmental requirements for ELYS in fertilized eggs of *Drosophila*

Despite the impact of both the morphology and dynamics of pronuclei on live birth outcome in assisted human reproduction (Azzarello *et al.* 2012), little is known about pronuclear coordination. We showed that, in *Drosophila*, the maternally provided ELYS protein has an important functional role in pronuclear apposition. Although the precise localization patterns of ELYS at the female and male pronuclei deep within an embryo remain ambiguous, its localization at the nuclear rim and the interior of the nucleus has been shown in *Drosophila* S2 cells (Doronin *et al.* 2024). Pronuclear localization of ELYS is common in many animal species (Sutovsky *et al.* 1998; Fernandez and Piano 2006; Galy *et al.* 2006; Inoue and Zhang 2014; Cavazza *et al.* 2021). In *Caenorhabditis elegans*, maternally provided MEL-28, the homolog of ELYS, is required for the structural integrity of the nuclear envelope (Fernandez and Piano 2006; Galy *et al.* 2006), which is necessary for pronuclear migration depending on the cargo-transport mechanism involving the LINC (linker of nucleoskeleton and cytoskeleton) complex (Padilla *et al.* 2022). However, when ELYS amount was decreased or increased experimentally in *Drosophila* embryos, cellular abnormalities became evident only at the step of pronuclear apposition following the migration of the pronuclei (Williams *et al*. 1997). Thus, the mechanisms underlying pronuclear migration and apposition appear to vary among organisms. The present study provides a novel role of ELYS that is essential during the binuclear stage at the beginning of *Drosophila* embryonic development. It would be interesting to investigate whether the ELYS function in pronuclear apposition is evolutionarily conserved.

The ELYS protein, one of the subunits of the Nup107-160 subcomplex, also known as the Y-complex, of the NPC, serves as a key constituent of the NPC scaffold (Hampoelz *et al.* 2019a; Huang *et al.* 2023). The main function of ELYS is to initiate the stepwise assembly of NPCs in late mitosis; chromatin-bound ELYS recruits the Nup107-160 subcomplex as nuclei reform (Archambault *et al.* 2022; Liu and Hetzer 2022; Capelson 2023). Although NPCs mediate essential cellular functions, the loss-of-function alleles of *Elys* in *Drosophila* (Hirai *et al.* 2018) and those of *mel-28* in *C*. *elegans* (the homolog of *Elys*; Gönczy *et al.* 1999) result in female sterility or maternal-effect lethality of the embryonic progeny produced by otherwise normal females. In assembling NPCs (Penzo and Palancade 2023), ELYS is only involved in the pathway that is active during late mitosis but not during interphase (Doucet *et al.* 2010; Funakoshi *et al.* 2011; Vollmer *et al.* 2015) or in the endoplasmic reticulum within structures referred to as annulate lamellae (Rasala *et al.* 2008). As annulate lamellae, containing numerous NPCs, are prominent in early *Drosophila* embryos (Hampoelz *et al.* 2016; Hampoelz *et al.* 2019b) and can contribute to the postmitotic formation and expansion of the nuclear envelope (Ren *et al.* 2019), this complementary mechanism may help explain why *Elys* loss-of-function mutants are viable in *Drosophila*.

**Fig. 4.**
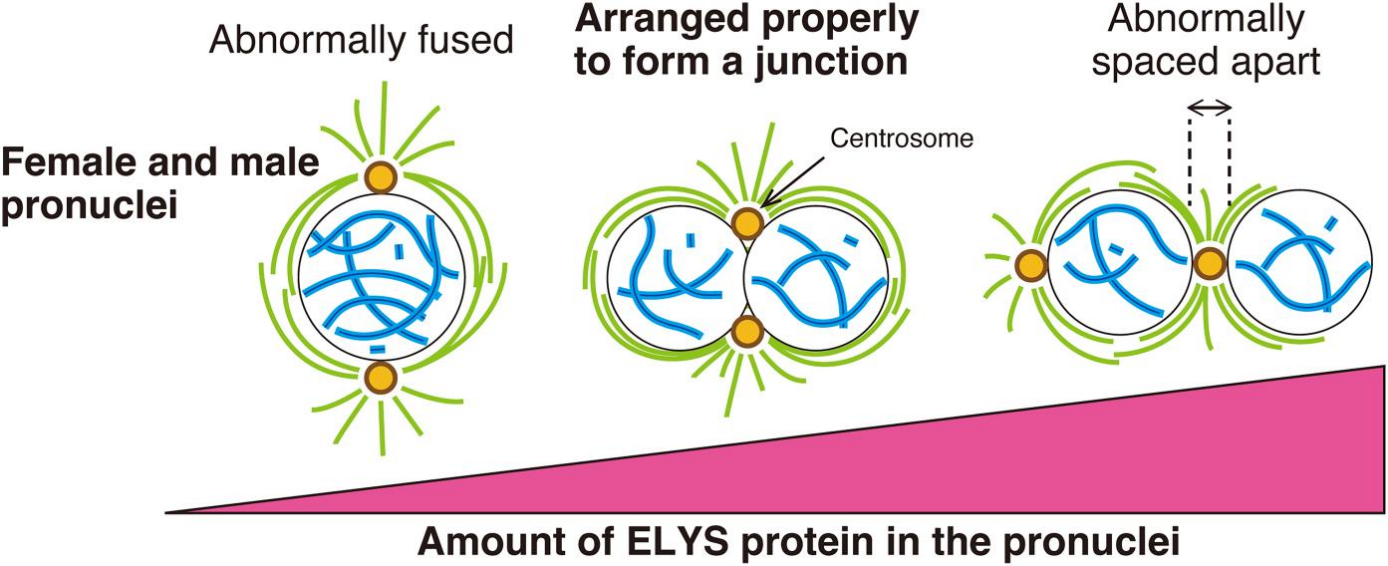
Model for pronuclear apposition at fertilization in *Drosophila*. The model depicts different configurations of the female and male pronuclei and the two centrosomes during the first mitotic prophase, depending on the amount of maternally provided ELYS localized in the pronuclei. Proper pronuclear apposition allows centrosome positioning at the pronuclear junction (center), ensuring correct bipolar spindle formation around two abutted sets of parental chromosomes for the first mitosis. The pronuclei that are abnormally fused (left) or spaced apart (right) result in faulty mitotic spindles.

A deficit of maternally provided ELYS caused abnormal conjugation of parental sets of chromosomes in fertilized eggs, whereas overexpressing ELYS resulted in an increase in the spacing between the two parental chromosome sets (Figs. 2 and 3). These two experimentally induced phenotypes suggest that, for normal pronuclear behavior leading to successful zygote formation, the level of ELYS needs to be optimal (Fig. 4). The effect of excess ELYS in fertilized eggs is reminiscent of that induced by the *Drosophila* maternal-effect mutation *fs(1)Ya* (Lin and Wolfner 1991; Liu *et al.* 1995). The YA protein localizes to the nuclear lamina and nucleoplasm of pronuclei and governs pronuclear apposition following migration (Lopez *et al.* 1994; Sackton *et al.* 2009). These results imply that interactions between pronuclei depend on the unique organization of the nuclear envelope specific to the pronuclear stage. ELYS is known to localize to the nuclear, but not cytoplasmic, side of NPCs (von Appen *et al.* 2015; Tai *et al.* 2022; Huang *et al.* 2023; Doronin *et al.* 2024). Factors like ELYS and YA in the interior of the pronuclei may regulate the developmental stage-specific functions of NPCs or other physical properties of the nuclear envelope, leading to proper pronuclear apposition by preventing their fusion or excessive separation. In bovine zygotes, NPCs drive the two interconnected processes of fertilization, namely the transport of pronuclei along the sperm aster and parental chromosome clustering at the pronuclear interface (Cavazza *et al.* 2021). Such parental genome clustering is not evident in *Drosophila*, but ELYS in the nuclear interior could have an impact on interactions between NPCs and microtubules involving dynein to achieve pronuclear apposition.

### Centrosome positioning at the pronuclear junction possibly serves as a basis to establish spindle bipolarity during the first mitosis

Centrosomes generally provide the mitotic spindle poles and direct spindle bipolarity (Hoffmann 2021; Hannaford and Rusan 2024). Following migration of the female pronucleus along the sperm aster, centrosome movement to opposite sides of the apposed pronuclei is important for proper spindle formation for the first mitosis (Blake-Hedges and Megraw 2019). We showed that, during the first mitotic prophase of the wild-type embryo, one of the two centrosomes, both of which originally accompany the male pronucleus, localizes to one side of the pronuclear junction and the other either at the opposite side of the junction or somewhere else on a pronucleus (Fig. 3, a and b). We infer that one centrosome may first be stabilized at the pronuclear junction and the other could move apart toward the opposite side to form the two poles of the first mitotic spindle. We might have captured a migrating centrosome in about half of the control embryos (Fig. 3b).

When ELYS was overexpressed, the embryos showed incomplete pronuclear apposition during prophase (Fig. 3c), commencement of mitotic spindle assembly around the pronuclei that are spaced apart during prometaphase (Fig. 3d), and a tripolar or deformed mitotic spindle during metaphase (categories 4 and 5 in Fig. 2a). It is not known whether the lack of pronuclear contact led to the abnormal spindle formation or not. However, it is a likely possibility that, in fertilized eggs, pronuclear apposition and centrosome positioning at the junction establish an initial scaffold that precedes assembly of a proper bipolar mitotic spindle for the first mitosis (Fig. 4). In early *Drosophila* embryos, both centrosomal and acentrosomal spindle assembly mechanisms are operational (Sonnenblick 1950; Komma and Endow 1997; Dix and Raff 2007; Blachon *et al.* 2014; Hayward *et al.* 2014; Dubruille *et al.* 2023; Hirai *et al.* 2023). Accordingly, upon ELYS overproduction, bipolar microtubule arrays were organized not only around the pronucleus associated with two centrosomes but also around the other pronucleus with only one shared centrosome (as shown in the image for category 4 in Fig. 2, a and b). The microtubules emanating from a centrosome and kinetochore fibers form parallel overlaps involving dynein for spindle pole focusing (Goshima *et al.* 2005). Thus, the distinctive tripolar first mitotic spindle could have formed with the central poles connected by a centrosomal aster and the distal poles that remain separate. We presume that the normal bipolarity of the first mitotic spindle, which consists of two units of microtubule arrays, might depend on the centrosomes that reside at or around the opposite sides of the pronuclear junction during prophase, refining spindle pole organization (Fig. 4).

In the abbreviated cell cycle of early *Drosophila* embryos (Foe *et al.* 1993; Farrell and O'Farrell 2014), centrosome duplication normally occurs in late mitosis, and the two centrosomes at each spindle pole do not separate until late telophase (Rothwell and Sullivan 2000). When ELYS is missing or overexpressed, precocious separation of two centrosomes and their subsequent dissociation from the spindle poles during prometaphase–metaphase were often detected in embryos (categories 3 and 5 in Fig. 2). As ELYS did not localize to centrosomes (Figs. 1 and 3), the protein probably has no direct influence on centrosome dynamics or positioning. Instead, abnormal centrosome behavior could result from any spindle defects, DNA damage stress, or impaired activity of cytoplasmic dynein (Archambault and Pinson 2010). Centrosomal defects might be responsible for complete dissociation of two microtubule arrays of a first mitotic spindle (as shown in the images in category 5 of Fig. 2, a and b).

### Possible involvement of ELYS and Nup160 in hybrid incompatibility

In the *Elys*^+^  *D*. *melanogaster* genetic background, ELYS^sim^ overexpression resulted in maternal-effect lethality of the embryonic progeny (Supplementary Table 3), showing incomplete pronuclear apposition and tripolar or deformed spindle formation (Supplementary Table 4; Fig. 2l), as is the case with ELYS^mel^ overexpression (Fig. 2h). In addition, when ELYS^sim^ was expressed in *Elys*^−^  *D*. *melanogaster* females, the embryo also formed such abnormal tripolar spindle (Fig. 2j). Moreover, as we have shown previously (Hirai *et al.* 2018), this phenotypic consequence is also the case for the ectopic expression of the *D*. *simulans* version of Nup160, one of the interacting subunits of ELYS within the Nup107-160 subcomplex of NPCs (Bilokapic and Schwartz 2013; Bui *et al.* 2013; von Appen *et al.* 2015; Tai *et al.* 2022). In other words, the phenotype of the *Nup160*^sim^ introgression is similar to that of the gain-of-function, not loss-of-function, of *Elys*. We assume that the ELYS^sim^-Nup160^mel^ and ELYS^mel^-Nup160^sim^ interactions—as well as an increased level of ELYS^mel^ or ELYS^sim^—all cause the fertilization failure with the same mechanistic basis in *D*. *melanogaster* embryos. Further research is needed to determine the molecular basis of the incompatible interactions between interspecific ELYS and Nup160. It also will be of interest to explore the possible specie-specific coevolution of the nucleoporins as it potentially contributes to hybrid incompatibility.

## Supplementary Material

jkaf104_Supplementary_Data

## Data Availability

Strains and reagents generated in this study are available upon request. The authors affirm that all data necessary for confirming the conclusions of the article are presented within the article, figures, and tables. More information can be found in the Supplementary material. Supplemental material available at *G3* online.
