# Supplementary material for: The Drosophila nucleoporin ELYS is required for parental chromosome arrangement at fertilization
Source: G3 (Bethesda). 2025 May 13;15(7):jkaf104. doi: 10.1093/g3journal/jkaf104 (PMC12239628; doi:10.1093/g3journal/jkaf104)
Supplement: jkaf104_Supplementary_Data [file jkaf104_supplementary_data.zip › Supplementary_Figure_Legends_G3-2025-405714.docx]

**Supplementary Fig. 1.**

Results of qRT-PCR. Repeated data and the mean are shown graphically. a) Difference between the effect of the *matα-Gal4* and *nos-Gal4* drivers on transgenic *Elys*^mel^-*mCherry* expression. b) Expression of transgenic *Elys*^mel^-*mCherry* with the *matα-Gal4* driver relative to the endogenous *Elys*^+^. The transgenic *Elys*^mel^ is not expressed without a driver in *Elys*^+^; *Elys*^mel^-*mCherry* females. The following primers were used for PCR amplification. For the control *Rp49*, 5´-TTC CTG GTG CAC AAC GTG-3´ and 5´-TCT CCT TGC GCT TCT TGG-3´. For *Elys*^mel^-*mCherry*, 5´-CCC GAC TAC TTG AAG CTG TCC-3´ and 5´-GTA GAT GAA CTC GCC GTC CTG-3´. A part of the mCherry sequence fused to the transgene allele was amplified. For endogenous *Elys*^+^ vs. exogenous *Elys*^mel^, 5´-TGG ATG CCG TTA AGA GAG CTC-3´ and 5´-ATC AGT TCG CAG AGG AAT GGG-3´ (b). The target sequence is in exon 7 of *Elys*, the 5´-side half of which is deleted in *Elys*^5^. Therefore, there is no PCR amplification for the *Elys*^5^ allele (*Elys*^-^).

**Supplementary Fig. 2.**

Results of qRT-PCR for *Elys* transgenes from *Drosophila* *melanogaster* and *D*. *simulans*: effect of drivers. Repeated data and the mean are shown graphically. a) Comparing the expression of *Elys*^mel^-*mCherry* vs. *Elys*^siml^-*mCherry* with the *matα-Gal4* driver. b) Comparing the expression of *Elys*^mel^-*mCherry* vs. *Elys*^sim^-*mCherry* with the *nos-Gal4* driver. The following primers were used for PCR amplification. For the control *Rp49*, 5´-TTC CTG GTG CAC AAC GTG-3´ and 5´-TCT CCT TGC GCT TCT TGG-3´. For *Elys*^mell^-*mCherry* and *Elys*^siml^-*mCherry*, 5´-CCC GAC TAC TTG AAG CTG TCC-3´ and 5´-GTA GAT GAA CTC GCC GTC CTG-3´. A part of the mCherry sequence fused to the transgene alleles is amplified.

**Supplementary Fig. 3.**

Localization of ELYS at initial stages of fertilization. Embryos produced by wild-type females of *Drosophila* *melanogaster* were fixed and stained with the DNA dye DAPI and an antibody specific for ELYS. (a) Immediately after completion of female meiosis. The schematic diagram of the anterior of a newly fertilized egg shows the sperm nucleus in the center of the egg and four products of female meiosis that align perpendicularly to the egg cortex. The area marked with the square corresponds in position to the adjacent confocal images. ELYS is detectable at all four products of female meiosis (the innermost, arrow; the remaining three, outlined arrows) but not at the sperm nucleus (arrowhead). Insets show a 3× magnified view of the innermost female meiotic product and the sperm nucleus. (b) During pronuclear migration. In the schematic diagram, the area marked with the square corresponds in position to the adjacent confocal images. The innermost female meiotic product becomes the female pronucleus (arrow) and migrates toward the male pronuclei (arrowhead). ELYS is localized to both the female and male pronuclei. Scale bars, 10 μm.

**Supplementary Fig. 4.**

Localization of ELYS during the first and second mitotic divisions in *Drosophila simulans* embryos. *Drosophila simulans* embryos were fixed and stained as described in Fig. 1. a) Pronuclear apposition. The pronuclei are arranged, forming a junction. ELYS labeling is faint in the nucleoplasm. b) First metaphase. The spindle consists of two halves of microtubule arrays, each encompassing a parental set of replicated chromosomes. ELYS is broadly distributed between the poles in each microtubule unit. c) First anaphase. ELYS broadly localizes to the entire spindle area between the poles. d) First telophase. ELYS localizes to daughter nuclei containing both parental sets of separated chromatids. e) Second metaphase. ELYS broadly localizes to the entire spindle area between the poles. f) ELYS is not detectable around the polar bodies. Scale bars, 10 μm.
