# Supplementary material for: The Drosophila nucleoporin ELYS is required for parental chromosome arrangement at fertilization
Source: G3 (Bethesda). 2025 May 13;15(7):jkaf104. doi: 10.1093/g3journal/jkaf104 (PMC12239628; doi:10.1093/g3journal/jkaf104)
Supplement: jkaf104_Supplementary_Data [file jkaf104_supplementary_data.zip › Supplementary_Tables_G3-2025-405714.docx]

Supplementary Table 1

Development of embryos produced by *Elys*^2^ females expressing *Elys*^mel^ transgenes

| Maternal genotype | | Hatchability (%) |
| --- | --- | --- |
| *Elys* locus | Transgene |  |
| *Elys*^2^ | *nos*-*Gal4* > *Elys*^mel^ | 9.5 |
| *Elys*^2^ | *nos*-*Gal4* > *Elys*^mel^-*mCherry* | 0.5 |
| *Elys*^2^ | *matα*-*Gal4* > *Elys*^mel^ | 39.5 |
| *Elys*^2^ | *matα*-*Gal4* > *Elys*^mel^-*mCherry* | 59.5 |

Females of the indicated genotypes were crossed with wild-type males, and egg hatchability was examined (n = 200, excepting *Elys*^2^; *matα*-*Gal4* > *Elys*^mel^-*mCherry*, n = 195). *Elys*^2^: one of the functionally null *Elys* alleles. The driving effect is significantly larger in *matα-Gal4* than in *nos-Gal4*: *Elys*^2^; *nos-Gal4* > *Elys*^mel^ vs. *Elys*^2^; *matα-Gal4* > *Elys*^mel^ (χ^2^ = 47.047, d.f. = 1, *p* = 6.931E-12) and *Elys*^2^; *nos-Gal4* > *Elys*^mel^-*mCherry* vs. *Elys*^2^; *matα-Gal4* > *Elys*^mel^-*mCherry* (Fisher’s exact test, *p* < 2.2E-16).

Supplementary Table 2

Development of embryos produced by *Elys*^–^ females expressing *Elys*^sim^ transgenes

| Maternal genotype | | Hatchability (%) |
| --- | --- | --- |
| *Elys* locus | Transgene |  |
| *Elys*^5^ | *nos*-*Gal4* > *Elys*^sim^ | 26.5 |
| *Elys*^5^ | *nos*-*Gal4* > *Elys*^sim^-*mCherry* | 10.5 |
| *Elys*^2^ | *nos*-*Gal4* > *Elys*^sim^ | 5.5 |
| *Elys*^2^ | *nos*-*Gal4* > *Elys*^sim^-*mCherry* | 11.0 |
| *Elys*^5^ | *matα*-*Gal4* > *Elys*^sim^ | 1.5 |
| *Elys*^5^ | *matα*-*Gal4* > *Elys*^sim^-*mCherry* | 7.5 |
| *Elys*^2^ | *matα*-*Gal4* > *Elys*^sim^ | 15.0 |
| *Elys*^2^ | *matα*-*Gal4* > *Elys*^sim^-*mCherry* | 6.5 |

*Drosophila* *melanogaster* females of the indicated genotypes were crossed with wild-type males, and egg hatchability was examined (n = 200).

Supplementary Table 3

Overproduction of ELYS^sim^ is toxic to embryonic development

| Maternal genotype | | Hatchability (%) |
| --- | --- | --- |
| *Elys* locus | Transgene |  |
| *Elys*^+^ | *nos*-*Gal4* > *Elys*^sim^ | 96.0 |
| *Elys*^+^ | *nos*-*Gal4* > *Elys*^sim^-*mCherry* | 91.5 |
| *Elys*^+^ | *matα*-*Gal4* > *Elys*^sim^ | 2.0 |
| *Elys*^+^ | *matα*-*Gal4* > *Elys*^sim^-*mCherry* | 5.5 |

*Drosophila* *melanogaster* females of the indicated genotypes were crossed with wild-type males, and egg hatchability was examined (n = 200). Hatchability was significantly reduced with *matα-Gal4* compared to *nos-Gal4*: *Elys*^+^; *nos-Gal4* > *Elys*^sim^ vs. *Elys*^+^; *matα-Gal4* > *Elys*^sim^ (Fisher’s exact test, *p* < 2.2E-16) and *Elys*^+^; *nos-Gal4* > *Elys*^sim^-*mCherry* vs. *Elys*^+^; *matα-Gal4* > *Elys*^sim^-*mCherry* (χ^2^ = 292.67, d.f. = 1, *p* < 2.2E-16).

Supplementary Table 4

Incomplete pronuclear apposition and formation of abnormal first mitotic spindle in embryos produced by *Drosophila* *melanogaster* females overexpressing ELYS^sim^

| Maternal genotype | Percentage of embryos | | | |
| --- | --- | --- | --- | --- |
|  | Incomplete pronuclear apposition in prophase | | Tripolar or deformed first mitotic spindle in metaphase | |
| *Elys*^–^; *matα*-*Gal4* > *Elys*^sim^-*mCherry* | 80.0 | (n = 5) | 56.7 | (n = 30) |
| *Elys*^+^; *matα*-*Gal4* > *Elys*^sim^-*mCherry* | 80.0 | (n = 10) | 88.6 | (n = 35) |
