# Supplementary figures and images for: The Drosophila nucleoporin ELYS is required for parental chromosome arrangement at fertilization
Source: G3 (Bethesda). 2025 May 13;15(7):jkaf104. doi: 10.1093/g3journal/jkaf104 (PMC12239628; doi:10.1093/g3journal/jkaf104)

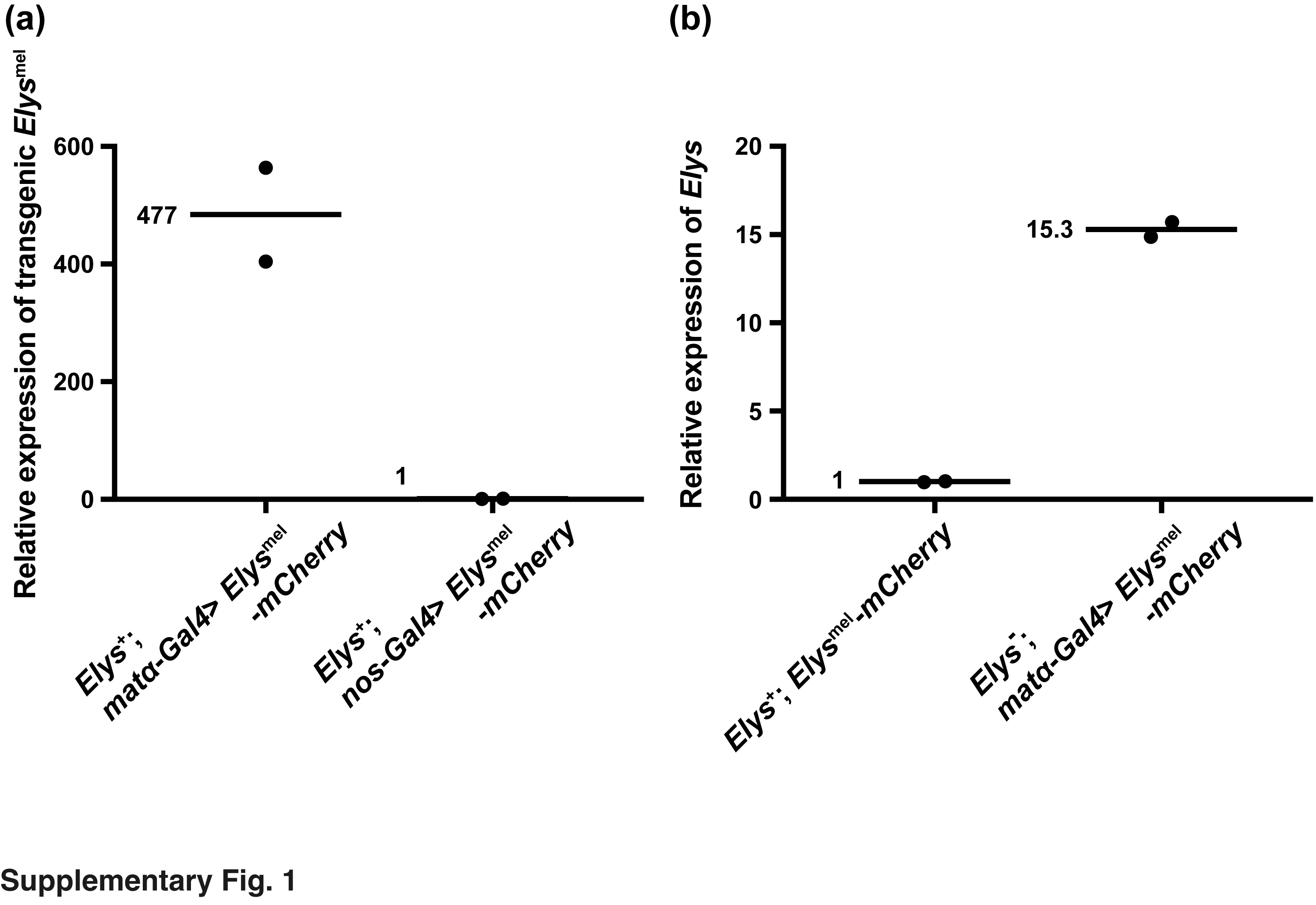

Supplement: jkaf104_Supplementary_Data [file jkaf104_supplementary_data.zip › Supplementary_Fig._1_G3-2025-405714.tif]

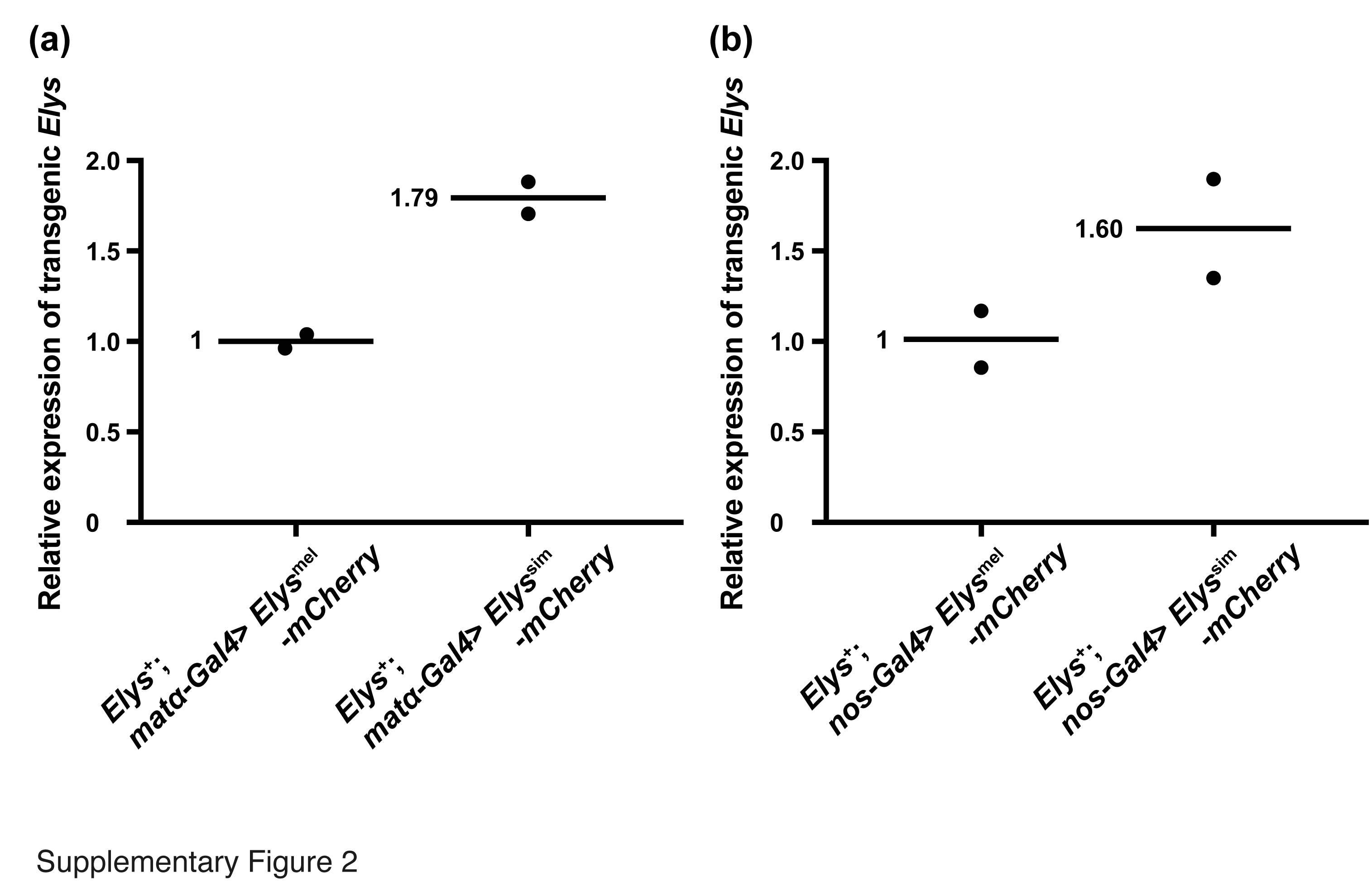

Supplement: jkaf104_Supplementary_Data [file jkaf104_supplementary_data.zip › Supplementary_Fig._2_G3-2025-405714.tif]

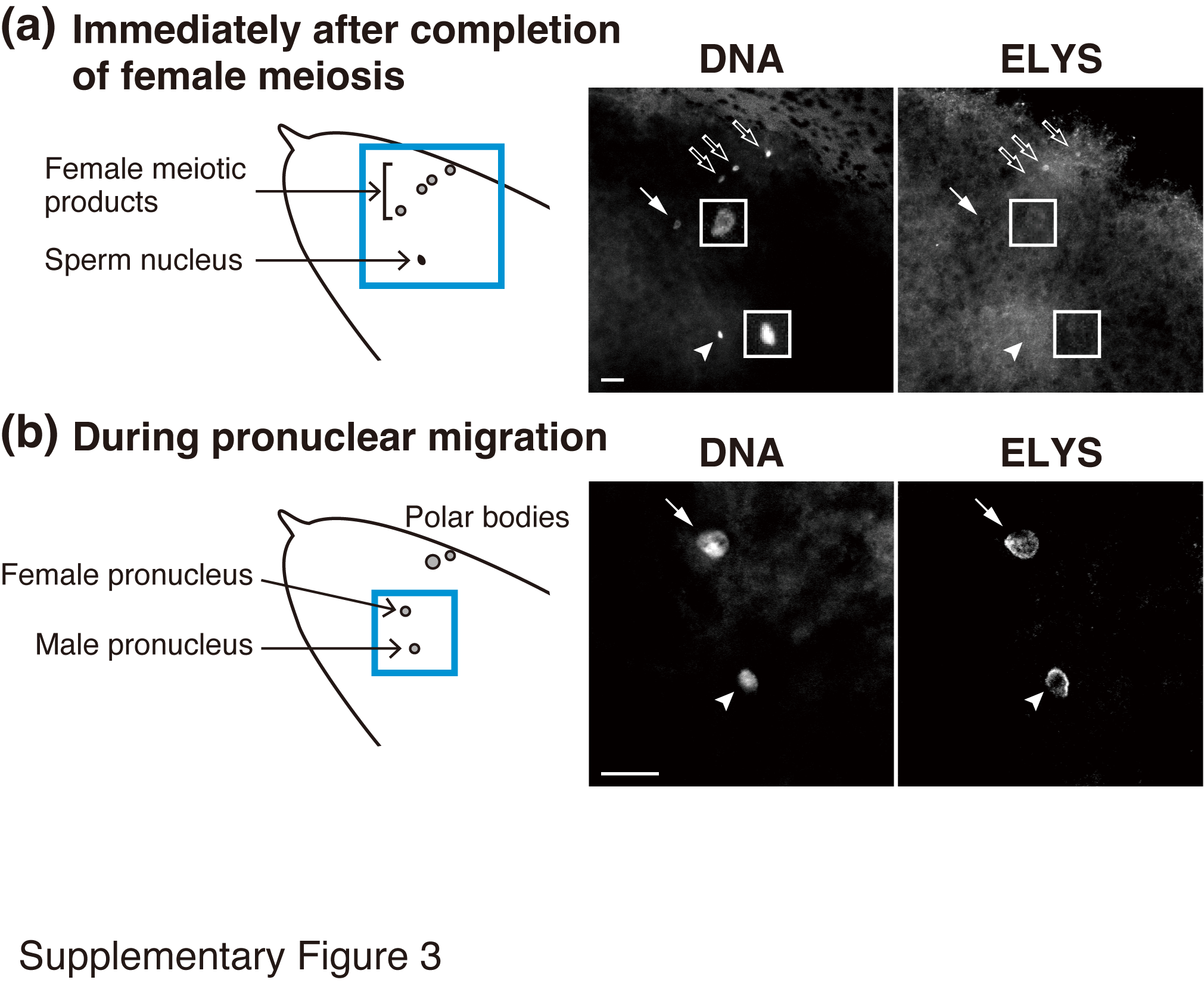

Supplement: jkaf104_Supplementary_Data [file jkaf104_supplementary_data.zip › Supplementary_Fig._3_G3-2025-405714.tif]

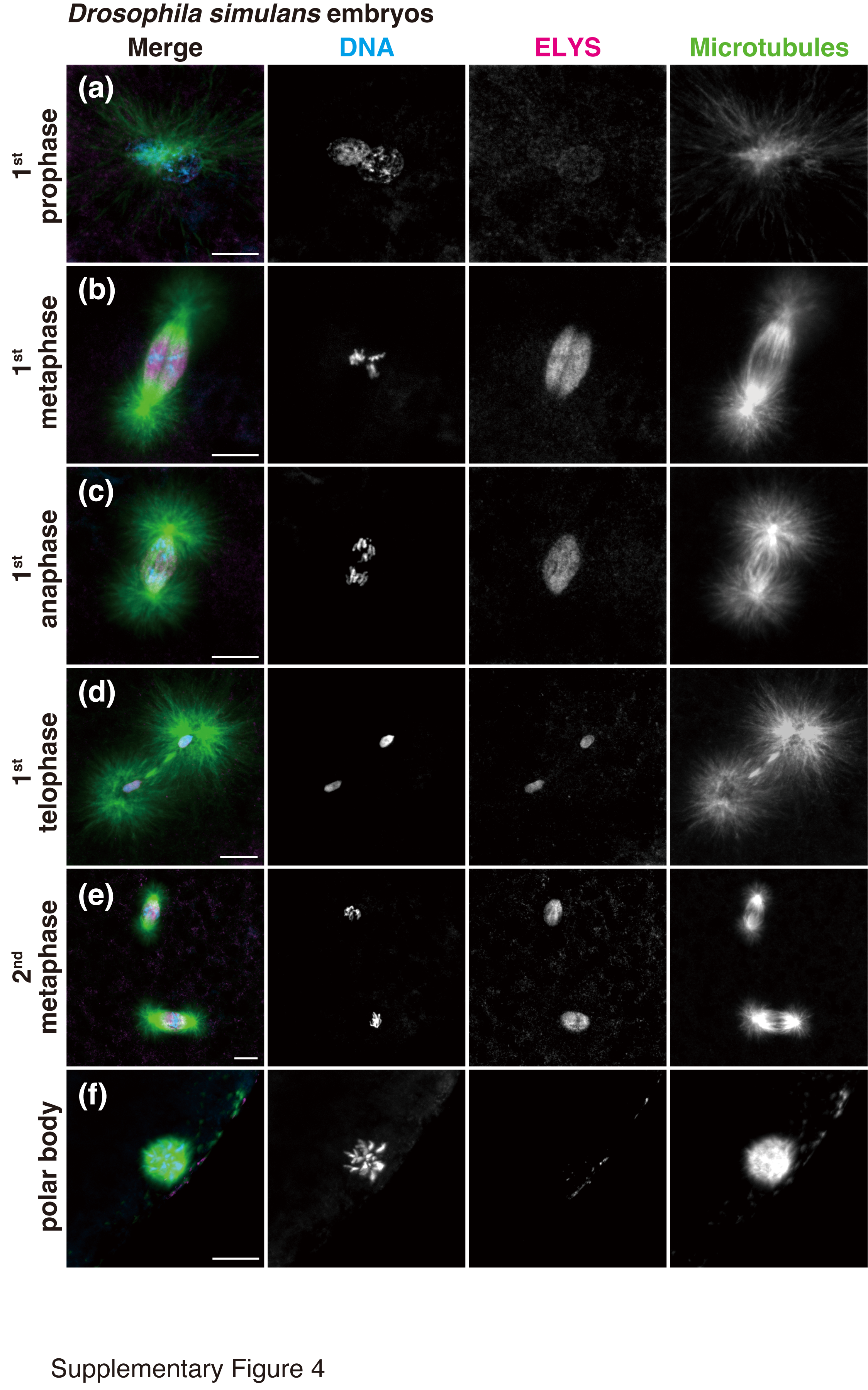

Supplement: jkaf104_Supplementary_Data [file jkaf104_supplementary_data.zip › Supplementary_Fig._4_G3-2025-405714.tif]
